# Persistent Bacterial Bronchitis: Time to Venture beyond the Umbrella

**DOI:** 10.3389/fped.2017.00264

**Published:** 2017-12-11

**Authors:** Andrew Bush

**Affiliations:** ^1^Imperial College London, London, United Kingdom; ^2^National Heart and Lung Institute, London, United Kingdom; ^3^Royal Brompton Harefield NHS Foundation Trust, London, United Kingdom

**Keywords:** cystic fibrosis, primary ciliary dyskinesia, endotype, airway infection, airway inflammation, bronchoalveolar lavage

## Abstract

Chronic cough in children is common and frequently mismanaged. In the past, cough was diagnosed as asthma and inappropriate asthma therapies prescribed and escalated. It has been realized that persistent bacterial bronchitis (PBB) is a common cause of wet cough and responds to oral antibiotics. The initial definition comprised a history of chronic wet cough, positive bronchoalveolar (BAL) cultures for a respiratory pathogen and response to a 2-week course of oral amoxicillin–clavulanic acid. This is now termed PBB-micro; PBB-clinical eliminates the need for BAL. PBB-extended is PBB-micro or PBB-clinical but resolution necessitating 4 weeks of antibiotics; and recurrent PBB is >3 attacks of PBB-micro or-clinical/year. However, the airway has only a limited range of responses to chronic inflammation and infection, and neutrophilic airway disease is seen in many other conditions, such as cystic fibrosis and primary ciliary dyskinesia, both chronic suppurative lung disease endotypes, whose recognition has led to huge scientific and clinical advances. There is an urgent need to extend endotyping into PBB, especially PBB-recurrent. We need to move from associative studies and, in particular, deploy sophisticated modern –omics technologies and systems biology, rather as has been done in the context of asthma in U-BIOPRED. In summary, the use of the term PBB has done signal service in pointing us away from prescribing asthma therapies to children with infected airways, but we now need to move beyond a simple description to teasing out underlying endotypes.

The history of our understanding and management of chronic cough in children is a litany of wrong diagnosis, inappropriate therapy, and muddled thinking. Cough was initially attributed to bronchitis, and treated with antibiotics; next it was appreciated that some children who coughed also had asthma, and responded to asthma therapy. This led to the catastrophic explosion of asthma mis-diagnoses (1–3), when normal children coughing with viral colds were diagnosed as asthmatic, and treatment escalated every time they coughed with another cold, despite ample evidence that escalating asthma therapy above low-dose inhaled corticosteroids is subject to the law of very rapidly diminishing returns (4). Subsequently, systematic studies (5) showed that the commonest cause of chronic wet cough was what was termed “persistent bacterial bronchitis” (PBB), many cases of which responded to a relatively short course of oral antibiotics. Noteworthy in this paper was that around 50% of these coughers were given an initial diagnosis of asthma, which was proven correct in less than 10%. The response to antibiotics was confirmed in a relatively small randomized controlled trial (6), which although a commendable attempt to secure an evidence base, used a cough score (7) as an end-point, rather than any more scientific biomarker or objective cough counting. The next consideration became the relationship between PBB and future bronchiectasis—would untreated PBB lead to bronchiectasis eventually, and would we, with this new paradigm of early and aggressive use of antibiotics prevent long term lung damage? I return to this important question below.

So, by going back to the future, have we made any progress? Of course, it is good that children with chronic airway infection are not treated with inhaled corticosteroids, which will likely worsen, not improve airway defenses (8–11), and to that extent the term PBB has served us very well. But it is now time to move beyond this, and this will only happen if weak thinking and general complacency about airway disease, and a culture where the so-called clinical diagnoses and assessments are deemed acceptable instead of making objective measurements is filtered out of the bloodstream of pediatric pulmonology where it is currently so strong. At present, there is little sign that this change is happening. Here, I advance the proposition that PBB is a description, not a diagnosis; and, in 2017, it is an umbrella term of exclusion. Failure to appreciate this will lead to scientific bankruptcy; PBB should be the start of thinking, not the finish. Elsewhere it has been argued that asthma is no more a useful diagnosis than arthritis or anemia (12), and I would argue the same is true for PBB. Chronic suppurative lung disease (CSLD) is well ahead of asthma in terms of defining airway endotypes, but still has a long way to go, as exemplified by PBB.

The aim of this annotation is to critically review our concepts of PBB, and where we should go next, especially in light of the recent ERS statement (13). The aim is to be provocative; so many airway diseases are stuck in thought-free ruts, and we need to lift our eyes to the towering achievements in those CSLDs which have moved out of the rut.

## What is PBB?

The initial definition was (i) a history of chronic wet cough, (ii) positive bronchoalveolar (BAL) cultures for a respiratory pathogen, and (iii) response to a 2-week course of oral amoxicillin–clavulanic acid (5). The definition has been modified, in part rightly reflecting the inappropriateness of bronchoscopy in many of these children (14). The original definition is now termed PBB-micro; PBB-clinical eliminates the need for BAL, and overtly acknowledges the need to exclude other causes of chronic wet cough, which is implied but not stated in PBB-micro. Unfortunately, there is no requirement to try to define infection non-bronchoscopically, with either cough swabs or better, induced sputum which is feasible even in very resource poor settings (15) and very young children (15, 16) and gives results comparable to BAL. This is a sad exemplar of the current “don’t measure” culture of pediatric pulmonology, which is a significant omission, as is the absence of any requirement to test if infection has resolved with antibiotics. The ERS statement (13) effectively defines PBB-clinical as PBB, but allows up to 4 weeks of antibiotics to resolve symptoms; again, there is no requirement for positive bacteriology. PBB-extended is PBB-micro or PBB-clinical but resolution necessitating 4 weeks of antibiotics; and recurrent PBB is >3 episodes of PBB/year. As to what investigations should be performed to exclude other causes of chronic wet cough, this is left to such energy and enthusiasm as may be possessed by the treating physician. It is perfectly clear that the infected airway signals a problem in stereotypic fashion—wet cough, respiratory distress, wheeze related to secretion retention—whether the cause be, for example, anatomical airway obstruction, a local or systemic immunodeficiency, or any one of many aspiration syndromes.

## What are CSLD Airway Endotypes?

An endotype is a subtype of a condition defined by a distinct pathophysiological mechanism (17). The march to CSLD endotypes started in 1938, when Dorothy Anderson first identified the pancreatic disease of cystic fibrosis (CF) (18), thus beginning the pathway to the differentiation of CF from other causes of chronic wet cough and bronchiectasis. A series of brilliant discoveries has led to the determination of the underlying gene defect (19–21) and the development of specific diagnostic tests [sweat test (22), genotyping (23), transepithelial potential differences (24, 25)] which make a diagnosis certain in all but the most difficult cases; and finally to diagnosis after newborn screening (26). The treatment has gone from non-specific therapies directed at the downstream consequences of *CFTR* dysfunction to designer molecules correcting the basic defect (27–29), and evidence for benefit has come from huge randomized, double-blind controlled trials. Finally, the age of personalized medicine in CF is dawning with the use of rectal spheroids (30) to determine *in vitro* the likely response to designer molecules *in vivo*.

Another obvious CSLD endotype is primary ciliary dyskinesia (PCD). From Kartagener’s original description (31) *via* Afzelius’ brilliant linking of electron microscopic abnormalities in sperm tails of infertile men with the syndrome (32), thus implicating ciliary dysfunction as the primary abnormality, the modern age has witnessed an explosion of diagnostic tests (33), including ciliary motility, electron microscopy [including electron microscopic tomography (34)], genotyping (35), and immunofluorescence of ciliary proteins (36). The intricacies of ciliary assembly are being unraveled, which has brought the realization that PCD can result not just from mutations in ciliary proteins but also in mutations in proteins responsible for ciliary assembly (37, 38). As yet therapeutic progress has lagged behind, although it is clear that some therapies which work well for CF (rhDNase) are useless or harmful in most PCD patients (39). The systemic immunodeficiencies are also being unraveled, with many specific genetic defects emerging from the umbrellas of antibody deficiency and common variable immunodeficiency (40).

In summary, the overwhelming message is that the discrimination of specific CSLD endotypes has led to an explosion of progress in the understanding of disease pathophysiology, and is likely to lead to novel and highly specific treatments, and which will not be possible unless endotypes are defined. Indeed, without defining endotypes, progress will not be made and valuable therapies discarded. So in asthma, if Brown had not shown that oral prednisolone only worked in wheezy patients with sputum eosinophilia, we would have lost that most efficacious of respiratory treatments (41). In later years, the anti-interleukin 5 monoclonal mepolizumab would have been discarded as ineffective (42) were it not to have been trialed in a specific group of asthmatics, with persistent airway eosinophilia and recurrent acute asthma lung attacks (43, 44). Also of note, without appreciating the different molecular subclasses of CF, the new molecules would probably have been discarded as useless if prescribed for all comers with the disease, let alone all those with a wet cough. So endotype-based therapy must be the target; where does this leave PBB?

## PBB Endotypes?

The broadening of the spectrum of PBB beyond a single episode (above) certainly implies different endotypes, and there are tantalizing hints from the literature that not all “PBB” is equal. A bad outcome (bronchiectasis) is associated with poor response to antibiotics (45), recurrent PBB, and isolation of *Haemophilus Influenzae* (46). Clearly a child with a single episode of chronic wet cough which responds to a single 2-week course of co-amoxyclav and never relapses requires no clinical investigation, although such children may be a useful control group to compare with those requiring multiple antibiotic courses and those who progress to bronchiectasis. The child needing prolonged and recurrent courses of antibiotics is not merely a diagnostic puzzle but also a scientific conundrum; what is the pathophysiology? The important question has been raised as to whether PBB is a precursor of bronchiectasis. The concept of a pre-bronchiectatic state has been proposed (47), and there is much robust evidence in favor of it. It is biologically inconceivable that airways can pass virtually instantaneously from normal caliber to fixed and irreversible dilatation. Indeed, it is clear both from immunodeficiency (48) and CF (49) that there is a phase of airway dilatation demonstrable on HRCT and which is reversible. It is also clinical experience that the progression of chronic airway infection to bronchiectasis can be halted if the underlying cause is remedied, for example, an endobronchial foreign body removed or chronic aspiration prevented. Hence, clearly for some patients with bronchiectasis, there will be a phase indistinguishable from PBB preceding airway dilatation, which likely (but unproven) may be reversed by intensive treatment.

It is clear that it would be unethical to do true natural history studies of chronic productive cough, to see who if untreated will progress to bronchiectasis and who eventually resolve their symptoms spontaneously. However, children who have had a prolonged, undiagnosed productive cough which eventually recovered undoubtedly exist in the community, never having been referred to secondary care, and should actively be sought as a control group. There are important questions we should start trying to address. First, does an episode of PBB-clin or -micro which responds to a 2-week course of antibiotics and never relapses represent a transient immunological insult, for example, a viral infection, which when overcome never recurs? Alternatively, are there genetic and epigenetic pathways which determine a benign, easily treated course despite an underlying mucosal immunodeficiency? If such a pathway existed and we understood it, might this open up new therapies for the more severe forms of the disease? Hypothetically, PBB could arise from infection with unusually virulent organisms (for which there is currently no evidence, but of course does not exclude the possibility that such evidence can be obtained) or an abnormal host response to common pathogens, and we need better to understand normal and pathological mucosal defenses. There are some such studies in the literature already (50–52), but we need to move from associative studies and, in particular, deploy sophisticated modern –omics technologies and systems biology, rather as has been done in the context of asthma in U-BIOPRED (53, 54). How can we predict who will progress to bronchiectasis? One approach would be to determine the immunological, molecular, and –omics signatures of established idiopathic bronchiectasis, and see if there is a group within the PBB umbrella in whom these signatures can be detected, implying they are the true pre-bronchiectatic PBBs. What are the biomarkers of progression, and conversely, of a response to treatment?

It could be questioned whether invasive approaches are ethical and appropriate in very young children. However, it should be noted that bronchoscopy, BAL lavage, endobronchial biopsy, and bronchial brushings are all acceptable diagnostic procedures in young children. I would argue that it is rather unethical to give prolonged and recurrent courses of antibiotics to young children without making every effort to establish the underlying diagnosis. Clearly, a single episode of PBB responding to a 2-week treatment course of oral antibiotics and never recurring does not merit invasive investigation, but how many courses, and for how long, would the pediatric community feel happy to administer “blind”? I would argue that currently we are too ready to sleep walk into more and more antibiotics without looking for specific diagnoses much more intensely.

## Twenty-First Century Treatment of PBB?

There is much scope for improvement of the current management of PBB, assuming that other underlying causes have been excluded. The presence of a wet cough and palpable secretions within the airway can be established clinically. However, whether this is due to bacterial infection cannot be, and every effort should be made to obtain lower airway cultures non-invasively. Second, cough and its frequency are poorly appreciated by parents and children (55) and we should surely objectively measure cough frequency over 24 h with one of a number of counters (56, 57). Validated questionnaires (7) are of course a step forward, but should not be a substitute for direct measurement. If antibiotics are prescribed, these steps should be repeated to ensure that infection has cleared. If there is relapse, it is essential to re-evaluate the diagnosis, as well as repeating the basic measurements (above). The point at which more detailed investigation, including bronchoscopy should be performed, and the role of airway clearance and mucolytics, particularly in preventing recurrence, needs to be explored. The pervasive “no-measurement” culture needs to be tackled firmly in PBB as elsewhere.

## Summary and Conclusion

It is clear that in the past, the umbrella term PBB has done signal service, not least in preventing the over-use of inhaled corticosteroids in children with a chronic wet cough. But it is also clear that it is only a staging post, and that we need to define PBB endotypes and move to specific treatments in particular in those who have PBB-extended and PBB-recurrent, including defining those at high risk of progressing to bronchiectasis. The time is right for a much more detailed and sophisticated assault on the underlying pathophysiology. Finally, we must avoid the treatment of PBB repeating the mistakes of history. Children were (and are) treated with inhaled corticosteroids for eosinophilic airway inflammation with no attempt to demonstrate that airway inflammation was actually present, and with bronchodilators without seeing if there was true reversible airway obstruction due to constriction of airway smooth muscle; too often we have given these treatments and believed the patient’s subjective impressions. This is akin to prescribing insulin without making any attempt to make measurements of blood glucose homeostasis. And yet all of the current PBB definitions include treatment with antibiotics, without mandating attempts to determine that (a) infection is present in the first place, (b) that infection has resolved, and (c) that cough frequency has returned to normal. This is not a tolerable standard of care in the twenty-first century. We should be looking at the dizzy heights reached in CF and move to emulate them in PBB.

## Author Contributions

The author confirms being the sole contributor of this work and approved it for publication.

## Conflict of Interest Statement

The author declares that the research was conducted in the absence of any commercial or financial relationships that could be construed as a potential conflict of interest.
